# Asymmetric impact of exchange rate on trade balance in Ethiopia: Evidence from a non-linear autoregressive distributed lag model (NARDL) approach

**DOI:** 10.1371/journal.pone.0311675

**Published:** 2024-12-05

**Authors:** Bazezew Endalew Abegaz

**Affiliations:** Department of Economics, College of Business and Economics, Bahir Dar University, Bahir Dar, Ethiopia; FUDMA: Federal University Dutsin-Ma, NIGERIA

## Abstract

Ethiopia has persistently pursued exchange rate devaluations to address its trade deficit and the structure of the economy, a strategy supported by the United Nations and economists. However, the effectiveness of this policy shift has sparked prolonged debate among scholars, exacerbated by divergent findings regarding the effect of exchange rates on trade balances. This study investigates the asymmetric effect of the real exchange rate on Ethiopia’s trade balance from 1992 to 2022. Employing the Non-linear Autoregressive Distributed Lag Model (NARDL), the research challenges the prevalent assumption of a linear (symmetric) association between exchange rates and trade balances in Ethiopian studies. Results reveal asymmetric effects of the exchange rate on the trade balance in both the short and long run, suggesting that exchange rate depreciations have varying implications for trade balances compared to appreciations of similar magnitude. Both real exchange rate depreciation and appreciation demonstrate statistically significant and positively asymmetric effects on Ethiopia’s trade balance across temporal dimensions. Specifically, a 1% exchange rate depreciation corresponds to a 0.843% and 0.856% improvement in the long-run and short-run trade balance, respectively. Furthermore, a 1% appreciation of the exchange rate is associated with a 15.079% and 15.02% enhancement of the long-run and short-run trade balance, respectively. The study underscores the importance of considering non-linear models of asymmetries in policymaking to inform more effective interventions.

## 1. Introduction

The success or failure of today’s economies, both at the national and institutional levels, hinges not only on their capacity to produce goods and services and the availability of resources but also on their competitiveness in international trade, especially in the context of an open economy. Consequently, optimal management of the exchange rate, in response to internal and external shocks, is deemed essential for maintaining economic stability and enhancing trade balance, as advocated by economists and institutions [1].

However, the relationship between exchange rates and trade balances has become a contentious topic among researchers in recent years. For instance, studies by [2–5] suggest that an increase in the exchange rate (depreciation) leads to an improvement in the trade balance. Conversely, empirical findings from [6–8] indicate that exchange rate depreciation leads to a trade deficit.

**Fig 1:**
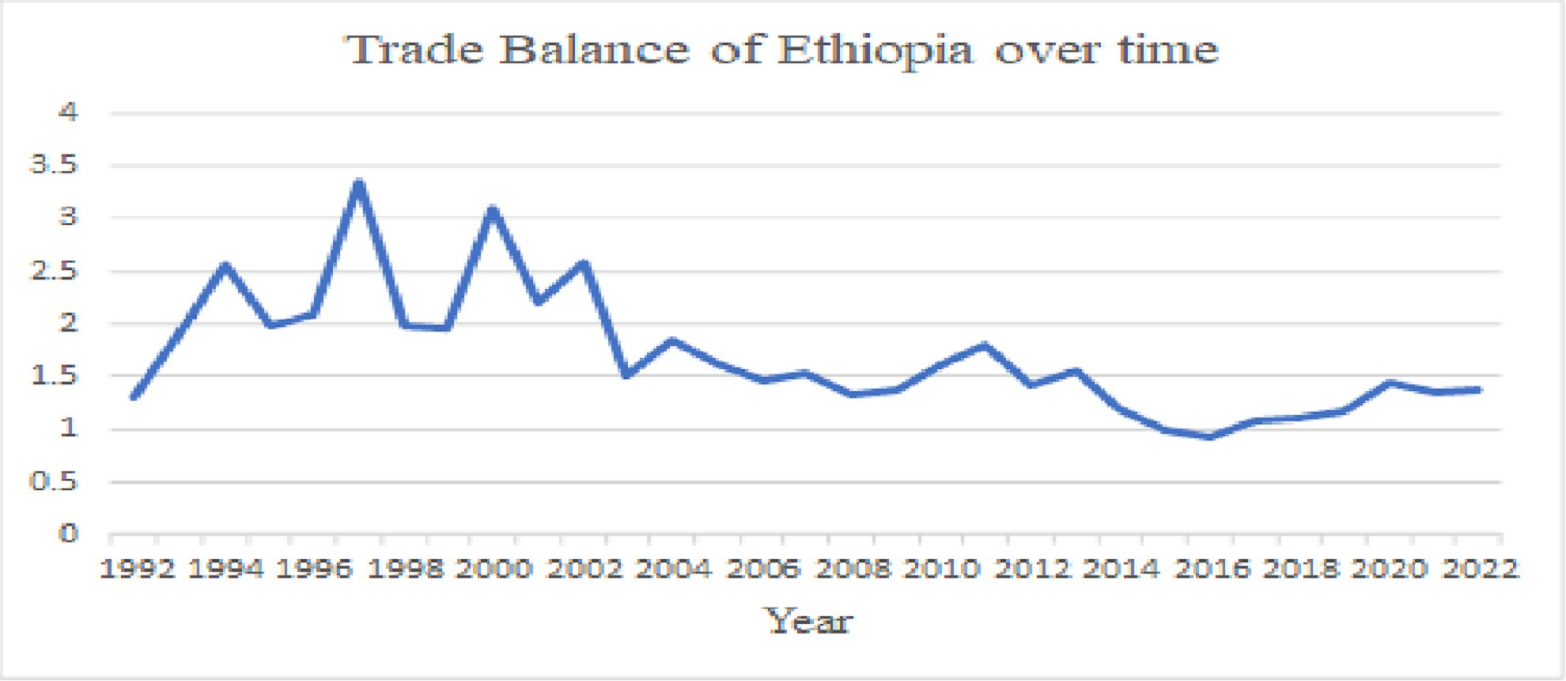
Trade balance of Ethiopia over time.

As we can observe from Fig 1, Ethiopia’s trade balance has not experienced sustained improvement over time. Structural issues such as constraints in export supply, competition primarily based on price rather than the quality of exported goods, challenges in substituting imports, unemployment, foreign currency-denominated debts, internal instability, and external shocks have been identified as key drivers of the problem [9, 10].

To tackle the problem, Ethiopia has implemented three chronic exchange rate devaluations against the US dollar since 1992, as the Ethiopian People’s Revolutionary Democratic Front (EPRDF) took power. During this time, the exchange rate of Ethiopia was undervalued by about 142%, as a result, the official exchange rate has been changed from 2.07 birr/dollar to 5 birr/dollar, which is considered to be the highest in the devaluation history of Ethiopia. The second one was started in September 2010 when it jumped from 13.6 birr/dollar to 16.3 birr/dollar, with a 16.7% devaluation rate, which was by far less than that of 1992. The last one was effective as of November 2017, changes the devaluation of birr against the US dollar by about 15% rate, as it was moved from 23.3 birr/dollar to 27 birr/dollar [9].

The prime objective of the devaluation undertaken at different periods was to maintain the trade balance through increasing export earnings. Exchange rate devaluation is expected to decrease the price of exported items of the small economy (Ethiopia) in the international market and increase the price of foreign goods and services imported to the devaluing country, this implies that the level of export increases, while that of imports decreases, lastly the economy resolves the imbalance/deficit problem. Under this circumstance, the basic issue is that the domestic economy (devaluating country) is expected to substitute the demand for imports, the consumption gap due to the high price of imports, and the economy would be in a position to enhance real output growth along with optimal utilization of existing resources [11–13].

**Fig 2:**
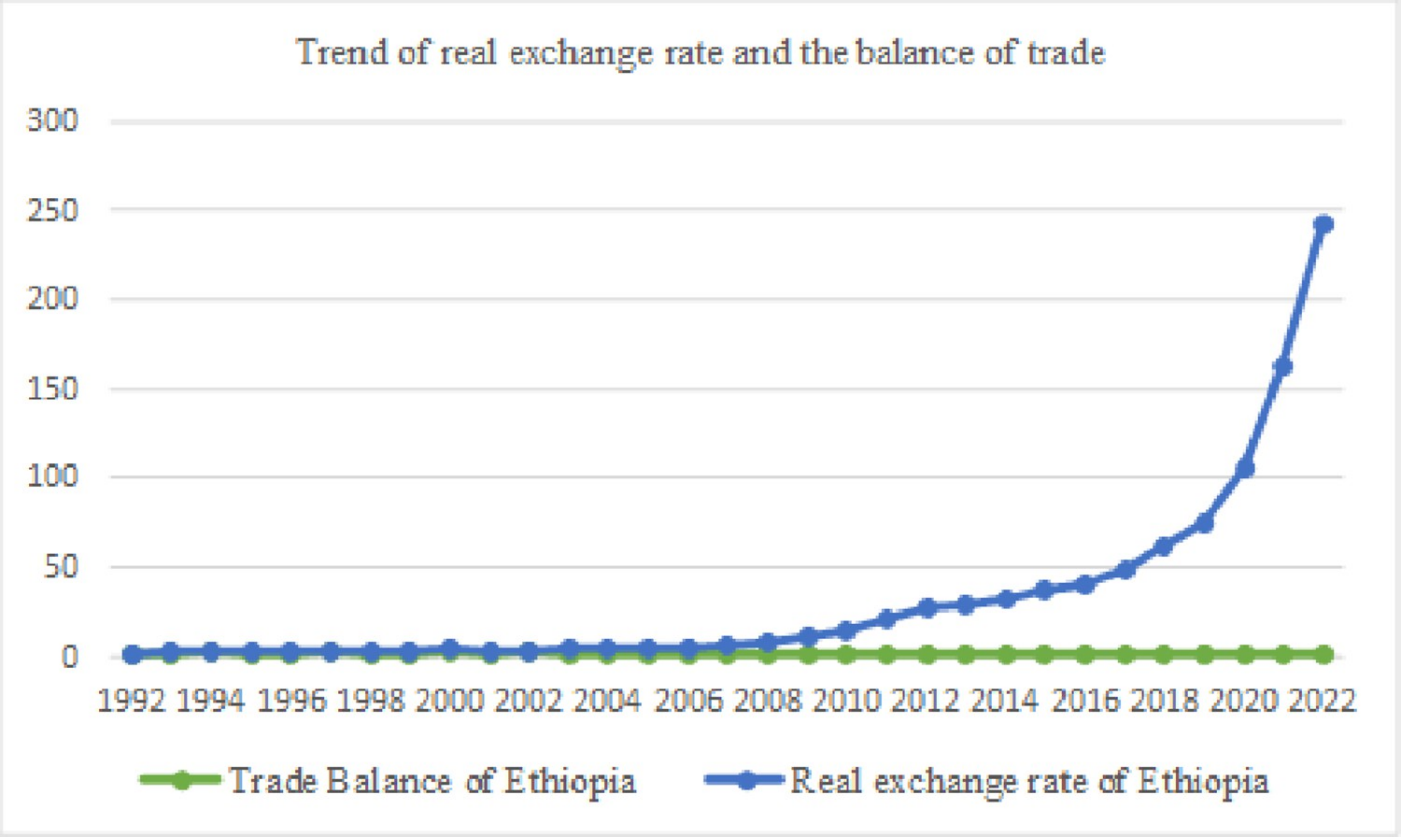
Trend of real exchange rate and the balance of trade.

However, as we can see from Fig 2, no significant change in the trade balance following a dramatic increment in the real exchange rate after 2007 was observed. Therefore, we are forced to suspect the existence of an exchange rate asymmetric effect in this economy because the Ethiopian economy is currently exposed to shocks and unforeseen events that the world experiences today.

The objective of this paper is to investigate the asymmetric effect of the exchange rate on the Ethiopian trade balance.

The basic contribution of this study to the current literature is twofold. The first major contribution lies in challenging the traditional assumption of symmetry in the relationship between exchange rates and trade balance. By recognizing that small economies like Ethiopia are often subject to unpredictable shocks and constraints, the study addresses the need to account for potential asymmetries in this relationship. This departure from linearity is important for providing a more accurate understanding of how exchange rate movements impact trade balance outcomes in Ethiopia. Utilizing the non-linear autoregressive distributed lag model (NARDL) allows the study to capture the potential asymmetric effects of exchange rate changes on the trade balance. This methodological approach is well-suited for analyzing the dynamics of exchange rate effects in the context of small, open economies, where traditional linear models may not fully capture the complexities of the relationship [6, 14].

Secondly, this study extensively incorporates basic theoretical foundations as a benchmark on the nexus of exchange rate and trade balance and extracts the other covariates of the model in addition to the variables of interest (exchange rate).

The remainder of the paper is structured as follows. Section two presents reviewed literature related to the inquiry. Section three presents the methodological setup of the study. Section four is devoted to the result and discussion parts. Section five presents concluding remarks and policy implications.

## 2. Literature review

### 2.1 Theoretical review

In real circumstances, the effect of exchange rate on the trade balance among countries varies following the variation of the socio-economic, regulatory systems, and institutional aspects of life, as a result, theories such as (a) the elasticity approach, (b) the Martial Lerner condition, (c) the J-curve phenomenon, (d) the monetarist approach, and (e) the Keynesian absorptive approach are adopted to explore the pass-through/channels of transmission between the exchange rate and trade balance extensively.

The elasticity approach asserts that the effect of the exchange rate on the trade balance of an economy depends on the extent of elasticity of demand for imported goods by domestic consumers and the demand elasticity of exported goods consumed by foreign consumers [15]. The basic idea behind this approach is that the degree of variation in the value and volume of export and import of goods and services matters to a change in trade balance. The theory predicts that if the demand elasticity of exported domestic goods by foreign consumers and imported goods by domestic consumers is elastic, then the economy’s trade balance is improved, otherwise, it results in a trade deficit.

To examine the mathematical version of the elasticity approach of exchange rate and trade nexus, we assume the small economy (countries that has no power to influence price in the market of the rest of the world) that makes the devaluation trades with the rest of the world. Moreover, domestic commodities imported from the rest of the world and exported to the rest of the world are imperfect substitutes for the small economy [13]. Therefore, the volume of imports demanded by the domestic economy,*M*_*D*_ and the quantity of imports demanded by the rest of the world, *M*_*F*_ are given in Eq (1) and (2) respectively as follows.


$$M_{D}=f(I_{D},P_{m},P),\frac{\partial M_{D}}{\partial I_{D}}>0,\frac{\partial M_{D}}{\partial P_{m}}<0,\frac{\partial M_{D}}{\partial P}>0$$ (1)

$$M_{F}=f(I_{f},P_{f},P^{*}),\frac{\partial M_{f}}{\partial I_{f}}>0,\frac{\partial M_{f}}{\partial P_{fe}}<0,\frac{\partial M_{f}}{\partial P^{*}}>0$$ (2)

Where, *I*_*D*_ is the domestic income level, *I*_*f*_ is the foreign income level, *P*_*m*_ is the domestic currency price of imported goods paid by domestic importers, while *P*_*f*_ is the foreign currency price level paid by foreign importers, *P* is the price of the entire domestically produced substitute goods in the small economy, while P* represents the price of the entire produced substitute goods of the rest of the world, and *e* is the nominal exchange rate. We further assumed that the commodities produced are normal goods (not inferior goods) and complimentary goods are not also considered since our data is aggregated in nature. As a result, both income elasticity and cross elasticity of demand are assumed to be positive, while that of own price elasticity of demand is negative for both domestic and foreign consumers.

For simplicity, the model assumes a homogenous demand function that the consumer is not affected by money illusion (demand remains the same even when money income and price double). This allowed us to modify the above equations and yield the real demand equations of real domestic income (*I*_*Dr*_) and real foreign income (*I*_*fr*_), we have also relative own domestic prices and foreign prices after adjustment, *RP*_*m*_ and *RP*_*f*_ respectively.


$$M_{D}=f(I_{Dr},{RP}_{m}),\frac{\partial M_{D}}{\partial I_{Dr}}>0,\frac{\partial M_{D}}{\partial {RP}_{m}}<0,\;I_{Dr}=\frac{I_{D}}{P}\;and\;{RP}_{m}=\frac{P_{m}}{P}$$ (3)

$$M_{F}=f(I_{fr},{RP}_{f}),\frac{\partial M_{f}}{\partial I_{fr}}>0,\frac{\partial M_{f}}{\partial {RP}_{f}}<0,\;I_{fr}=\frac{I_{f}}{P^{*}}\;and\;{RP}_{f}=\frac{P_{f}}{P^{*}}$$ (4)

According to [13], the relative price of imports (*RP*_*m*_) is equivalent to price of foreign exports interims of foreign currency adjusted for the exchange rate, hence, it is given by:

$${RP}_{m}=\frac{P_{m}}{P}=\frac{e{P^{*}}_{x}}{P}(\frac{{P^{*}}_{x}}{P^{*}})=ER\frac{{P^{*}}_{x}}{P^{*}}=RE(r{P^{*}}_{x})$$ (5)

Where P*_x_, is the foreign currency price of exports, *e* and *RE* are the nominal and real exchange rates respectively, and rP*_x_ is the real foreign currency price of exports.

The real exchange rate is the product of the nominal exchange rate (*e*) and the price ratios ($\frac{{P^{*}}_{x}}{P}$). The next basic component in the analysis of international trade is that of export. Therefore, the supply of exports by the domestic economy, *X*_*S*_ and the rest of the world, X*_s_ is given as follows.


$$X_{S}=f(P_{x},p)$$ (6)

$${X^{*}}_{s}=f({P^{*}}_{x},P^{*})$$ (7)

*P*_*x*_, Represents the domestic currency price of exports received by domestic exporters, whereas P*_x_ is the foreign currency price of exports received by foreign exporters. Finally, we are in a position to compute the balance of the trade equation at equilibrium as:

$$M_{D}=e{X^{*}}_{s}$$ (8)

$$M_{F}=X_{S}$$ (9)

Eq (9) states that domestic demand (import) of the small economy is equal to foreigners’ supply of exports adjusted for nominal exchange rate, similarly, foreign demand (import) is equal to the supply of exports by the small economy, here, no need of exchange rate adjustment since the small economy exporters are price takers in the market of the rest of the world. This implies that,

$$TB=P_{x}M_{F}-{(RE)(r{P^{*}}_{x})M}_{D}$$ (10)

Where, (*RE*) (rP*_x_) is the relative price of import defined in Eq (5), which is the product of the real exchange rate, *RE* and the real foreign price of exports received by foreign exporters,rP*_x_.

Ended, we have come up with the conclusion of the elasticity approach of predicting how the real exchange rate affects the balance of trade.


$$TB=f(I_{Dr},I_{fr},RE),\frac{\partial TB}{\partial I_{Dr}}<0,\frac{\partial TB}{\partial I_{fr}}>0,\frac{\partial TB}{\partial RE}>0$$ (11)

From Eq (11) we understand that when domestic real income increases, we expect domestic consumers to import more, and then the trade balance deteriorates and vice versa. On the other hand, if foreign real income increases, we expect import demand of the rest of the world will increase, this implies that the export of the domestic economy will increase and then the trade balance will be improved. Moreover, when the real exchange rate increases (depreciation of domestic currency), we expect high levels of export by the domestic economy and low levels of import, then the trade balance will be positive.

The other important component in the analysis of trade theory is the so-called Martial learner condition, it is just an extension of the elasticity approach postulates that if the sum of demand elasticity of import and export is greater than unity, then the trade balance is improved, provided that the trade balance is zero initially [16, 17].

The other analytical version of the elasticity approach is the J-curve phenomenon. According to the J-curve effect, currency devaluation increases the value of import in domestic currency and decreases the value of export in terms of foreign currency in the short-run, as a result, the volume of export increases and that of import decreases [16–18]. The implication is that the short-run and long-run effects of currency devaluation on the trade balance differ due to its volume and value effect. Export and import demands are inelastic in the short-run because of low or sluggish change in consumers’ preference and low bargaining skill of producers immediately to the devaluation, hence, the trade balance deteriorates in the short-run, while improved in the long run because of the elastic nature of import and export demands in the long-run.

However, the elasticity approach faced significant criticism, particularly on its partial analysis, it highly asserts the value and volume effect of the devaluation, which implies that it ignores the effect of the devaluation on the consumption and production level of the economy. This gap is explained by the Keynesian absorption approach.

According to the Keynesian absorptive approach, exchange rate devaluation affects the trade balance of an economy through its effect on aggregate consumption (absorption) and production (7). The model used consumption (C), investment (I), government expenditure (G), and import (M) as the basic components of the entire expenditure in an economy, the sum is generally called domestic absorption (A). Moreover, the entire income of an economy is composed of consumption, investment, government expenditure, and export (X). Thus, the aggregate expenditure (A) and income (Y) components are expressed mathematically in Eqs (12) and (13) respectively as follows.


A=C+I+G+M (12)

Y=C+I+G+X (13)

If we deduct Eq (12) from Eq (13), then we have a change in the trade balance equation equivalent to a change in aggregate income and consumption or absorption.


Δ(X−M)=Δ(Y−A) (14)

In conclusion, this Keynesian absorptive approach revealed that the trade balance is a function of national output and absorption. The currency devaluation improved the trade balance through its effect of increasing the aggregate output (Y) more than the aggregate absorption (A) [18, 19].

The last theoretical approach that explains the nexus between the trade balance and currency devaluation is the view of the monetarist [18, 20]. According to monetarists, the money market is the other channel of transmission between the exchange rate and trade balance. It argues that if money demand exceeds money supply, then the central bank increases the interest rate to reduce the demand for money so that the market of money becomes in equilibrium.

Foreign investors are coming to the domestic economy who are attracted by the higher interest rate, or there is an inflow of capital, this improves the trade balance, while a reduction in interest rate causes an outflow of capital and deteriorates the trade balance. The implication of this premise according to the monetarist is that exchange rate devaluation, which holds the money supply constant will decrease the balance of both real money demand and supply through its effect on price, implies that the domestic currency price of goods and services expected to be higher following a devaluation, interest rate will not increase, and capital inflow becomes lower. As a result, exchange rate devaluation deteriorates the trade balance, while its appreciation counterpart leads to improvement of the trade balance. The mathematical version of the monetarist view is, therefore,

$$TB=\frac{M^{s}}{P}=M^{d}(Y,Er)$$ (15)

Where *M*^*s*^ is money supply, *M*^*d*^ is money demand in the economy, *P* is the price level, *Y* is aggregate income, *Er* is the nominal exchange rate. The monetarists also revealed that the devaluation of the exchange rate by itself may improve the balance of trade in the short run by slugging the level of absorption following a reduction in real money demand as the price rises.

In conclusion, from the view of the monetarist, we understand that exchange rate devaluation has a dual effect on the trade balance. One is its negative effect on the trade balance through capital outflow due to the reduction of real money demand and then the level of interest rate. Secondly, it enhances the trade balance by reducing total absorption because of a reduction in real money demand [21–23].

The entire effect of exchange rate devaluation on the trade balance, therefore, depends on the net effect of exchange rate changes. If the level of capital outflow (reduction of aggregate output) following a reduction of interest rate outweighs the reduction of total absorption following lower real money demand, then the devaluation will have a deteriorating effect on the trade balance, whereas if the level of capital outflow (reduction of aggregate output) is less than the reduction of total absorption, then the devaluation is expected to improve the trade balance.

Ended, the study used the above theoretical frameworks (hypothesis) as a benchmark to incorporate the explanatory variables of the forthcoming model as well as to understand the pass-through effect of the exchange rate on the trade balance.

### 2.2 Empirical findings

According to the study of [6], the exchange rate has an asymmetric effect on the trade balance of Vietnam both in the short-run and long run, it confirmed that an increase in exchange rate decreases the trade balance, while the exchange rate appreciation of the same size with the depreciation does not affect the trade balance in the short-run. In the same country (Vietnam) [24], employed a panel co-integration approach and found that an increase in exchange rate and income deteriorates the trade balance, whereas foreign income positively affects the trade balance.

[15] investigated the effect of exchange rate on the trade balance of 51 advanced and emerging market economies and explored that exchange rate devaluation helps to adjust trade imbalance in these countries. Moreover [25], employed the NARDL model to investigate asymmetric effects of US exchange rate on its bilateral truism trade balance with Canada, Mexico and the UK. The finding revealed that US exchange rate depreciation improves its trade balance with all trading partners in the long-run, while the appreciation of US exchange rate deteriorates its long-run trade balance with Canada and the UK, but insignificant with Mexico.

[3] conducted a study on the asymmetric effect of the exchange rate on the trade balance of India, it indicated that appreciation deteriorates the trade balance, while depreciation of the exchange rate improves it in the short-run, a similar response is obtained in the long-run, but only the depreciation segment is statistically significant, whereas [26] investigated the asymmetric effect of exchange rate on the trade balance of Bangladesh and revealed that appreciation of exchange rate improves the trade balance. The symmetric assumption of exchange rate depreciation affects the bilateral trade of India and USA positively as it was investigated by [27]. The study of [28] employed an Autoregressive distributed lag model approach in the Cameroonian economy and found that exchange rate devaluation deteriorates the trade balance in the short run and improves in the long run, it confirms the prediction of the J-curve phenomenon. However, the findings of [29] show that an exchange rate devaluation yields an improvement of the trade balance both in the short-run and long-run in the economy of Albania, which proves the J-curve phenomenon did not occur. [28] investigated the nexus between real exchange rate and trade balance in ten Sub-Saharan Africa, it found that depreciation improves the trade balance of countries, except Senegal, Ghana, and Morocco.

Exchange rate depreciation impacts symmetrically the trade balance of Uganda [27]. This study confirmed that exchange rate depreciation deteriorate trade balance in the short-run, while it improves in the long-run. Similar result is investigated by [30] found out that exchange rate deprecation increases the trade balance. [31] investigated the nature of exchange rate dynamics and trade balance nexus in some selected African countries using both linear and non-linear models. The finding underscores that the linear model proves the existence of J-curve effect in the short-run only on Uganda, while in the long-run the J-curve holds only on Algeria. However, the non-linear model result confirmed that the short-run J-curve holds for both South Africa and Uganda, while the long-run J-curve holds on Algeria and Uganda. It concludes that utilization of asymmetric models are more efficient than linear counterparts.

On the other hand, currency depreciation does not affect the trade balance of Nigeria as revealed by the study of [32]

The study of [2] investigated the absence of the J- J-curve effect in the analysis of the exchange rate effect on the trade balance of Ethiopia by using the symmetric autoregressive distributed lag model. Specifically, it explores that exchange rate devaluation improves the trade balance both in the short run and long run. The study further disclosed that both domestic and foreign income improves the traded balance, whereas money supply and government expenditure negatively affect it. Another study conducted by [33] revealed that the positive or negative effect of exchange rate on trade balance of an economy is determined by both permanent and transitory external shocks, given the predetermined covariates. [34] reports the negative asymmetric effect of the depreciation on the trade balance of Chania and its trading partners. Differently [33], confirmed that the existence of transitory and permanent external shocks matters whether the effect of exchange rate devaluation on trade balance is positive or negative. Ended, inconclusive empirical results have been investigated by researchers so far. We explore the asymmetric effect of exchange rate change on the trade balance of Ethiopia by using the non-linear (ARDL) approach to contribute to the literature by using the theories discussed and the forthcoming analytical approaches extensively.

## 3. Methods and materials

### 3.1 The data

This study utilized secondary annual time series data from 1992 to 2022. The variables used in this study are trade balance (***TB***), real exchange rate (***RER***), foreign real gross domestic product (***FRGDP***), home real gross domestic product (***HRGDPC***), and government expenditure (***GX***). The foreign income (***FRGDP***) is computed by considering the average real gross domestic product of seven countries including, China, the United States of America, the United Kingdom, the United Arab Emirates, Saudi Arabia, Germany, and the Netherlands which are the major export and import destinations of Ethiopia over the last decades [2]. The total income of these countries are valued in terms of US dollar. The 32 years data for variables are meticulously sourced from the World Bank (WB). We present the details of the data in Table 1.

**Table 1:** Description of the data.

Data	Description	Unit	Source
Export volume index (2015 = 100)	It is the ratio of the export value indexes to the corresponding unit value indexes	__	World Bank
Import volume index (2000 = 100)	It is the ratio of the import value indexes to the corresponding unit value indexes	__	World Bank
Foreign consumer price index (2010 = 100)	Consumer price index, period average		World Bank
Foreign real gross domestic product	Constant 2015 US$	^____^	World Bank
Ethiopia Consumer price index (2010 = 100)	Consumer price index, period average	__	World Bank
trading partners consumer price index (2010 = 100)	Consumer price index, period average		World Bank
Gross domestic product per capita, constant prices	measured in constant price	The national currency, the Billion birr	World Bank
Nominal exchange rate	Read as 1 unit of US$ = units of Ethiopian birr.	US $/ETH birr	World Bank
General government total expenditure	Entire government expenditure	Billion Birr (local currency)	World Bank

### 3.2 Methods of data analysis

To analyze the data, we utilized a combination of both inferential (econometric) and descriptive techniques. For the former case, the study employed the non-linear autoregressive distributed lag (***NARDL***) model to estimate both the short-run and long-run asymmetric impact of the real exchange rate on the trade balance of Ethiopia along with the bound co-integration test approach.

For the latter, we employed straightforward statistical tools such as tables, graphs, and percentages to present a clear and concise representation of the data.

### 3.3 Econometric modeling and techniques of estimation

This section presents a detailed overview of the steps taken in our econometric modeling and estimation procedures, ensuring transparency and reliability in our analytical approach.

The basic model utilized to explore the asymmetric effect of the real exchange rate on the trade balance of Ethiopia is the non-linear autoregressive distributed lag model (***NARDL***). We employed this model for the following reasons. One is that the NARDL helps to test co-integration between model variables by itself in its bound approach [35]. Two, the NARDL model is preferred, since it provides efficient and reliable estimates in case of low and limited samples. The third important point is that the model provides both the short-run and long-run estimates simultaneously [6]. Finally, the model allows the variables to be integrated with different orders of integration, and estimated at different lag lengths in one regression model, this is, of course, the crucial feature of the NRADL.

To estimate the non-linear ARDL model, we decompose the variable of interest (real exchange rate) as negative and positive components; the negative part is an indication of the predicted appreciation effect of real exchange rate on the trade balance, while the positive part indicates the expected depreciation effect of real exchange rate on the trade balance, the model assumes the effects are asymmetric.

According to [36], the NARDL model is specified through the decomposition of the independent variable as its own negative and positive partial sums. The asymmetric effect of real exchange rate change on the trade balance in the NARDL approach of this study in natural logarithm form is specified as follows.


$${lnTB}_{t}=({{lnRER}^{+}}_{t},{{lnRER}^{-}}_{t},{lnFRGDP}_{t},{lnHRGDPC}_{t},{lnGX}_{t})$$ (16)

***TB***_*t*_: is the trade balance measured by the ratio of export volume to import volume at time t.

***RER***: is the real exchange rate of Ethiopia measured by the product of nominal exchange rate and ratio of domestic per foreign consumer price indexes (CPI). Following [2, 6, 37], the real exchange rate of Ethiopia is indicated in Eq (17) below.


$$RER=N_{Er}*\frac{P_{iH}}{P_{iF}}$$ (17)

***P***_***iH***_: is the price index (consumer price index of Ethiopia).

***P***_***iF***_: is foreign price indexes, it is the average consumer price index of the largest trading partners of Ethiopia in terms of export and import destinations [2] as we discussed in section 3.1. Consumer price index is utilized because it is a measure of competitiveness of the small economy with the rest of the world [37, 38].

*N*_*Er*_: Nominal exchange rate of Ethiopia expressed in USD/ETB. Therefore, increase in exchange rate means depreciation of exchange rate in Ethiopia. The decision to adopt the **US dollar** as the basis for measuring Ethiopia’s exchange rate stems from the fact that most international trade transactions are pegged to the dollar [39], additionally, exchange rate data for most countries, including Ethiopia, is commonly available relative to the US dollar through sources such as the **World Bank** and the IMF.

***RER***^+^_t_: is the expected positive (depreciation) effect of the real exchange rate on the trade balance at time t.

***RER***^−^_*t*_: is the expected negative (appreciation) effect of the real exchange rate on the trade balance at time t.

***FRGDP***_***t***_: Foreign income at time t.

***HRDP***_***t***_: is home real gross domestic product per capita at time t.

***GX***_*t*_: Government expenditure at time t.

The long-run and short-run NARDL estimations of the study as specified by [36] are given in Eqs (18) and (19) respectively as follows.


$${ln(TB}_{t})={€}_{0}+\sum_{i=1}^{q_{1}}\left({{€}^{+}}_{1}ln{{RER}^{+}}_{t-i}\right)+\sum_{i=1}^{q_{2}}\left({{€}^{-}}_{2}l{{nRER}^{-}}_{t-i}\right)+\sum_{i=1}^{q_{3}}\left({€}_{3}{lnFRGDP}_{t-i}\right)+\sum_{i=1}^{q_{4}}\left({€}_{4}{lnHRGDP}_{t-1}\right)+\sum_{i=1}^{q_{5}}\left({€}_{5}{lnGX}_{t-i}\right)+\sum_{i=1}^{q_{6}}\left({€}_{6}{lnTB}_{t-i}\right)+E_{t}$$ (18)

Where € **‘**s are long-run estimated parameters of the non-linear ARDL model,***q*’s** are the optimal lag length of the long-run model.


$${ln\Delta (TB}_{t})=\delta_{0}+\sum_{i=1}^{p_{1}}\left({\delta^{+}}_{1}\Delta ln{{RER}^{+}}_{t-i}\right)+\sum_{i=1}^{p_{2}}\left({\delta^{-}}_{2}\Delta l{{nRER}^{-}}_{t-i}\right)+\sum_{i=1}^{p_{3}}\left(\delta_{3}{\Delta lnFRGDP}_{t-i}\right)+\sum_{i=1}^{p_{4}}\left(\delta_{4}{\Delta lnHRGDP}_{t-1}\right)+\sum_{i=1}^{p_{5}}\left(\delta_{5}\Delta {lnGX}_{t-i}\right)+\sum_{i=1}^{p_{6}}\left(\delta_{6}\Delta {lnTB}_{t-i}\right)+E_{t}$$ (19)

Where, ***δ***’s are parameters of the short-run ARDL model, and ***p*** ‘s are optimal lags of the short-run model.

#### 3.3.1 Unit root test

For both the short-run and long-run non-linear ARDL models to be deemed reliable, the underlying variables must be stationary over time. This ensures that any shock to the outcome variable (*Y*_*t*_) is temporary and will dissipate over time. Traditional unit root tests, such as the Augmented Dickey-Fuller (ADF) test, assume that structural breaks in the time series are predetermined and occur due to one-time shocks. This can introduce biases when deciding whether to reject the null hypothesis of a unit root, as it may lead to incorrect conclusions about stationarity [40]. To address this, our study used the Zivot and Andrews unit root test [40] that allows for structural breaks that are endogenously determined by the data itself, rather than being fixed in advance. This approach is more flexible and can better capture breaks related to the data’s trend. In our unit root test, the null hypothesis posits that the data has a unit root with a trend, implying non-stationarity. The alternative hypothesis is that the data is stationary. If the Zivot and Andrews t-statistic exceeds the critical value at the chosen significance level, we reject the null hypothesis of non-stationarity with a trend, and thus accept the alternative hypothesis of stationarity.

#### 3.3.2 The nonlinear ARDL bound test of co-integration

To determine the long-run relationship between variables in the estimated model, the co-integration test is required. This study utilized the bound test approach to test the existence of co-integration in this study. To run the co-integration test, we develop the null hypothesis that there is no co-integration between variables in the non-linear ARDL model of Eq (18). If the result of the F-statistics value of the estimated bound test is greater than the critical values at 5%, then we reject the null hypothesis of no co-integration between the outcome variable and the regressors. Finally, we proceed to estimate and interpret the short-run and long-run asymmetric effects of the NARDL models as specified in Eqs (18) and (19).

#### 3.3.3 Diagnostic tests

We further need to verify the estimated model parameters are efficient and reliable enough for interpretation and forecasting. To do so, basic diagnostic tests such as tests for heteroscedasticity, autocorrelation, normality, model specification tests, and reliability tests are undertaken.

## 4. Results and discussions

### 4.1 Unit root test

Before estimating a certain model consisting of a time series data set, it is better to verify whether the sires have a unit root or not, because if the series is not stationary, it means that the regression results are spurious even if the relationship between explanatory variables and the outcome variable is significant in principle. Therefore, this study employed the Zivot and Andrews unit root test to run the unit root test. To do so, we are going to compare the Zivot and Andrews’s t-statics value of the NARDL model with the respective critical values at a 5% significance level both at the level and first difference forms. The hypotheses for the unit root test are as follows:

**Null Hypothesis (*H***_***o***_**):** The variables have a unit root with a structural break in both the intercept and the trend.

**Alternative Hypothesis (H**_**1**_**):** The variables do not have a unit root (i.e., they are stationary).

If the t-statistic is greater than the critical value at the 5% significance level (in absolute terms), we reject the null hypothesis that the variable has a unit root (non-stationary). In such cases, we accept the alternative hypothesis that the data is stationary. As observed in Table 2, the variables used in the model are stationary at their first difference I (1), except for the dependent variable, trade balance (***LNTBV***), and government expenditure (***LNGX***). For instance, the t-statistic for the positive component of the real exchange rate ***LNRER***^+^) is -3.533512. This value is less negative than the critical value of -5.08 at the 5% significance level, meaning we fail to reject the null hypothesis that the positive component of the real exchange rate is not stationary at the level. However, at the first difference, the t-statistic of -5.89542 is more negative than the critical value of -5.5 at the 5% level, allowing us to reject the null hypothesis and conclude that the positive component (depreciation) of the real exchange rate is stationary at the first difference. We apply the same fashion to interpret the remaining.

**Table 2:** Zivot and Andrew unit root test results.

Variables	At level		At 1^st^ difference		
	Constant and trend		Constant and trend		
	T-test	Cr. val(5%)	T-test	Cr. val (5%)	Order
LNTBV	-5.286688^*^	-5.08	-6.231	-5.67	I (0)
*LNRER* ^*+*^	-3.533512	-5.08	-5.895^**^	-5.5	I (1)
*LNRER* ^*-*^	-2.687921	-4.93	-5.012^**^	-4.98	I (1)
L*NHRGDPC*	-2.763185	-5.08	-5.166^**^	-5.10	I (1)
*LNFRGDP*	-4.954549	-5.08	-5.897^**^	-5.23	I (1)
LNGX	-5.328511^*^	-4.93	-5.726	-5.068	I (0)

* indicates the variables are stationary at the level form of 5% significance level, while

** indicates the variables are stationary at the first difference of 5% significance level.

### 4.2 The nonlinear ARDL bound test for co-integration

Checking for co-integration is a crucial step, especially in time series analysis. Co-integration implies a long-run relationship among variables, which is essential for understanding their dynamic interactions over time. The presence of co-integration allows for meaningful interpretation of the relationships and enhances the reliability of long-term forecasts. As we can observe from Table 3, the NARDL bound test confirmed that the F-statics value (7.356553) is greater than the upper bound critical values of 4.68 at a 1% significant level. This enables us to reject the null hypothesis of no co-integration between the dependent and independent variables, this implies that there is a co-integration (long-run) relationship.

**Table 3:** Results of the NARDL F- bound test.

F-statistics: 7.356553*		
K = 5		
Critical values	Lower bound	Upper bound
10%	2.26	3.35
5%	2.62	3.79
1%	3.41	4.68

K: indicates the number of regressors

* indicates the co-integrated nature of the variables of the model at a 1% level of significance.

Once, the regression model confirms the existence of a long-run relationship, the next step is the estimation of the long-run and short-run asymmetric effect of the NARDL model result.

### 4.3 Long-run asymmetric effect of the NARDL model

The long-run results of the study are presented in Table 4. The results indicate that the depreciation of the Ethiopian real exchange rate (***LNRER***^+^) has an asymmetric and statistically significant positive effect on the trade balance in the long run at the 5% significance level. Specifically, holding all other factors constant, a 1% depreciation of the Ethiopian birr relative to the US dollar improves Ethiopia's trade balance by approximately 0.843431%. Conversely, the appreciation of the Ethiopian real exchange rate (***LNRER***^−^) also has an asymmetric and significant positive effect on the trade balance at the 1% level. In this case, holding other factors constant, a 1% appreciation of the Ethiopian birr relative to the US dollar improves Ethiopia’s trade balance by about 15.07979%. The likely reason that exchange rate appreciation improves trade balance is that an appreciated currency lowers the cost of importing goods, particularly if a country imports a large volume of essential raw materials or capital goods used in production. If cheaper imports lead to lower production costs for domestic industries, this could enhance their competitiveness globally, improving the trade balance in the long run. The second possibility is that if the demand for exports is relatively inelastic (i.e., foreign buyers continue purchasing despite higher prices) and the demand for imports is elastic (i.e., domestic buyers reduce purchases when imports become cheaper), appreciation could have a positive effect on the trade balance.

**Table 4:** Long-run results of the NARDL model: Dependent variable trade balance.

Variables	Coefficients	Standard errors	P-value
LNRER^+^	0.843431	0.399052	0.0462**
LNRER ^-^	15.07979	3.557108	0.0011*
LNHRGDPC	2.008189	0.939899	0.0539
LNFRGDP	-4.291062	1.922147	0.0454**
LNGX	-1.116921	0.441483	0.0264**
Constant	-163.7445	55.56106	0.0122**

** indicates variables are statistically significant at a 5% level, while

*indicates variables are statistically significant at a 1% level.

R-squared 0.9446

Prob(F-statistic) 0.00020*

The positive long-run effect of the depreciation of the Ethiopian real exchange rate on the trade balance is compatible with the study of [6] on the Vietnam economy, although this study reveals no long-run asymmetric effect of the appreciation of Vietnam real exchange on its trade balance. The findings of this study is partly consistent with the study of [2] who revealed that exchange rate depreciation improves the trade balance of Ethiopia in the long-run symmetrically, this implies that appreciation of exchange rate deteriorates Ethiopian trade balance in the long-run by the same level of the depreciation effect. Moreover, the study result of [41] who investigated US trade imbalances and east Asian exchange rates show that devaluation of US exchange rate had a positive symmetric effect not only on US trade balance but also its trading partners of east Asian countries (because according to the researcher devaluation of US $ reduce the local currency cost of imported oil, commodities, food and imported inflation of these countries), it supports this study result, given the symmetric assumptions. The study result contradicts the findings of [42] who revealed that both exchange rate appreciation and depreciation have significant and negative long-run asymmetric effects on the trade balance of some African economies. Moreover, unlike this study result, the utilization of linear and non-linear models yields no co-integration between exchange rate and trade balance according to the study of [43]. Ended, exchange rate volatility can improve the trade balance of an economy as investigated by [44].

The study’s finding that government expenditure *(**LNGX**)* has a significant and negative effect on Ethiopia’s trade balance in the long run at a 5% significance level suggests that increased government spending leads to a deterioration in the trade balance. Specifically, for every 1% increase in government expenditure, the trade balance worsens by approximately 1.1169%. The probable reason is that higher government expenditure, especially on infrastructure or public services, can stimulate demand for imported goods, worsening the trade balance, in addition to this, large government expenditures can crowd out private investment, leading to lower export growth or competitiveness in international markets, further impacting the trade balance [18]. Similar study assured this long-run negative association between the two [2, 45]. However, the study of [46] investigates that government expenditure improves the trade balance.

The study’s finding that foreign income *(**LNFRGDP******)*** negatively affects Ethiopia’s trade balance in the long run at a 5% significance level adds another dimension to the challenges faced by the country’s trade balance. Specifically, it indicates that for every 1% increase in foreign income, the Ethiopian trade balance deteriorates by approximately 4.2911%. This negative effect is explained by the substitution effect which examines when foreign income rises, those countries are able to shift away from importing goods and services from smaller economies like Ethiopia. Instead, they may focus on domestic production or import from more developed markets, which in turn reduces demand for Ethiopian exports, especially for non-diversified economies that heavily rely on a limited set of export commodities [9]. This result is supported by [47] who disclosed that foreign income increment reduce trade balance in West Africa economies and monetary union in the long-run. However, the result contradict with the findings of [2, 37, 48] showed that increase in foreign income improves the trade balance because foreign income improvement may increase the demand of their imports and, hence, export of small economy increases, then the trade balance will increase.

However, the effect of home income *(**LNHRGDPC***) has not statistical significant effect on trade balance of Ethiopia in the long-run at 5% significant level.

### 4.4 Short-run results

The short-run coefficients of the non-linear ARDL model indicate the short-run relationship between the trade balance and other covariates and it is presented in Table 5. The result of the study shows that the depreciation of the real exchange rate has a positive and significant short-run asymmetric effect on the trade balance of Ethiopia at a 1% level both at level form and its first lag. It indicates that other things remain the same, on average, a percent depreciation of the real exchange rate at level (Δ**LNRER**^+^**)** improves the trade balance by 0.8567% in the short run, while the first lag of the depreciation (Δ***LNRER***^+^(−**1**)) improves the trade balance by about 0.234535% in respond to a percent increase in exchange rate in the short-run. The implication is that the relatively high elasticity of the trade balance to the exchange rate depreciation in the short term suggests that Ethiopia’s trade responds quickly to changes in the exchange rate. This could be due to the export sector’s sensitivity to price competitiveness or the country’s dependence on imports. It is similar to the result of studies conducted in Ethiopia [2, 49], whereas authors [50, 51] investigated the negative short-run symmetric effect of exchange rate on the trade balance. Moreover, the appreciation of the real exchange rate has a positive and significant short-run asymmetric effect at a 1% significant level (Δ***LNRER***^−^). Specifically, it shows that other things are the same, on average, a percent appreciation of the real exchange rate leads to a 15.0231% improvement in the trade balance in the short-run.

**Table 5:** Short-run results of the model.

Variables	Coefficients	Standard errors	P-value
**COINTEQ(-1)**	-0.139411	0.017630	0.0000*
ΔLNRER^**+**^	0.8567	0.211986	0.0018*
ΔLNRER^+^ (-1)	0.234535	0.2134565	0.0023*
ΔLNRER ^**-**^	15.0231	1.900873	0.0000*
ΔLNRER—(-1)	-2.708360	0.903782	0.0111**
ΔLNHRGDPC	2.008189	0.541765	0.0030*
ΔLNFRGDP	-4.291061	1.408571	0.0102**
ΔLNFRGDP(-1)	-8.580202	1.694205	0.0003*
ΔLNGX	-0.215546	0.215855	0.5213
**constant**	-163.7445	20.73754	0.0000*

** indicates variables are statistically significant at a 5% level, while

*indicates variables are statistically significant at a 1% level

R-squared 0.903222

Prob(F-statistic) 0.000001*

However, the appreciation of the real exchange rate at its first lag (Δ***LNRER***^−^(−**1**)**)** has a negative and statistically significant asymmetric effect on the trade balance in the short run at a 5% level. All else the same, on average, a percent appreciation of the real exchange rate of Ethiopia relative to US dollar leads to a 2.70836% deterioration of the trade balance. The result is consistent with the findings of [42] who investigated that both appreciation and depreciation of exchange rate improve the trade balance of some African economies in the short run, except the first lag of the appreciation effect. It contradicts the findings of [52] on its appreciation case and reveals the asymmetric effect of exchange rate on cross-border trade in the Nigerian economy, the study disclosed that appreciation of exchange rate deteriorates trade, while depreciation improves the trade balance. Another author [53] investigated that the exchange rate has an asymmetric effect on the trade balance of US economy both in the short and long run. Moreover [34], reports the negative asymmetric effect of the depreciation on the trade balance of China and its trading partners.

The other variable that positively and statistically affects the trade balance in the short run is domestic real gross domestic product per capita (Δ**LNHRGDPC)** at a 1% levels of significance. It indicates that all else the same, on average, a percent increase in domestic economic growth leads to a 2.008189% improvement of the trade balance. As domestic per capital income rises, the country may develop import-substitution industries. These industries produce goods locally that were previously imported, reducing the overall import bill. As domestic production increases and the economy becomes more self-sufficient, imports decrease, improving the trade balance. This result is supported by a study conducted in Ethiopia [2], but the study of [47] revealed dichotomous result on West African economies and disclosed that domestic income has positive effect on the trade balance of Benin and Senegal, but negative in case of Burkina Faso, while [37] reports negative effect of domestic income on the Kenyan trade balance in the short-run.

Once again foreign income of trading partners negatively and significantly affects trade balance of Ethiopia in the short-run at 5% and 1% level of significance both at level **(**Δ**LNFRGDP)** and first lag **(**Δ**LNFRGDP**(−**1**)) respectively. The result contradict with [2, 37, 47] that investigates short-run improvement of trade balance following foreign income increment in the short-run.

The error correction term (ECT) plays a crucial role in measuring how quickly a system returns to its long-run equilibrium after a shock or deviation. Once the existence of co-integration between the dependent variable and its covariates is established, the ECT provides insight into the speed of this adjustment. Therefore, the lagged error correction term **(**Δ**COINTEQ**(−**1**)) is statistically significant at a 1% significant level. It shows that all else is the same, if the trade balance has deviated from its long-run equilibrium in the last period, then 13.94% of the deviation is corrected in the current year. Thus, it would take approximately seven years and one month for an entire (100%) adjustment of shocks to reach its stable long-run equilibrium.

### 4.5 Structural stability and diagnostic tests

Testing diagnostics and structural stabilities are crucial to verify the reliability and optimality of the regression coefficient. As is presented in Table 6, the basic diagnostic tests incorporated in this paper are the Breusch-Godrey Serial Correlation LMTest, the Breusch/Pagan test for heteroscedasticity, the Ramsey RESET test of correct model specification, and the Jarque-Bera test on normality. As the result indicates, there is no problem with autocorrelation (serial correlation) since we failed to reject the null of no serial correlation because the p-value (0.5690) is greater than the margin error of 5% (0.05). Similarly, the residuals have common variance and constant mean over the sample periods as we confirmed that there is no problem of heteroscedasticity. The Ramsey RESET test indicated that the model is correctly specified because we already accept the null hypothesis because the p-value of (0.2523) is greater than 0.05. The study result also confirms the residuals are normally distributed. Overall, the diagnostic tests confirm that the model is free from issues such as serial correlation, heteroscedasticity, model misspecification, and non-normal residuals. This supports the validity, reliability, and robustness of the estimated regression coefficients.

**Table 6:** Diagnostic test results.

Model diagnostic	T- stat.	p-value	decision
Breusch-Godrey Serial Correlation LMTest:	0.596895	0.5690*	failed to reject Ho
*Ho*: *No serial correlation*			
Breusch/Pagan heteroskedasticity test	0.947031	0.3402*	failed to reject Ho
*Ho*: *no heteroscedasticity problem*:			
Ramsey RESET test (F)	1.9685	0.2523*	failed to reject Ho
*Ho*: *the model is correctly specified*:			
Jarque-Bera test on normality (chi2)	1.0354	0.3521*	failed to reject Ho
*Ho*: *the residuals are normally distributed*:			

*: refers statistically significant at 5%

**Fig 3:**
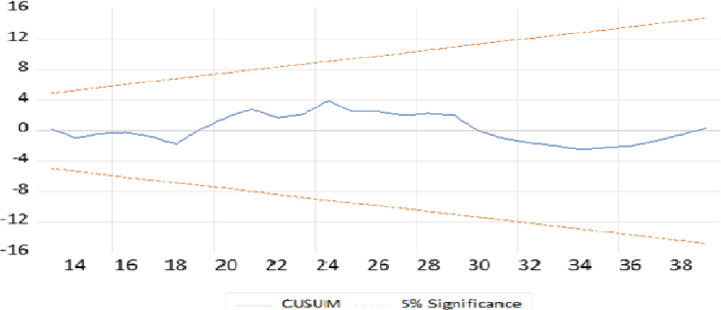
Cumulative sum of recursive residuals. CUSUM.

**Fig 4:**
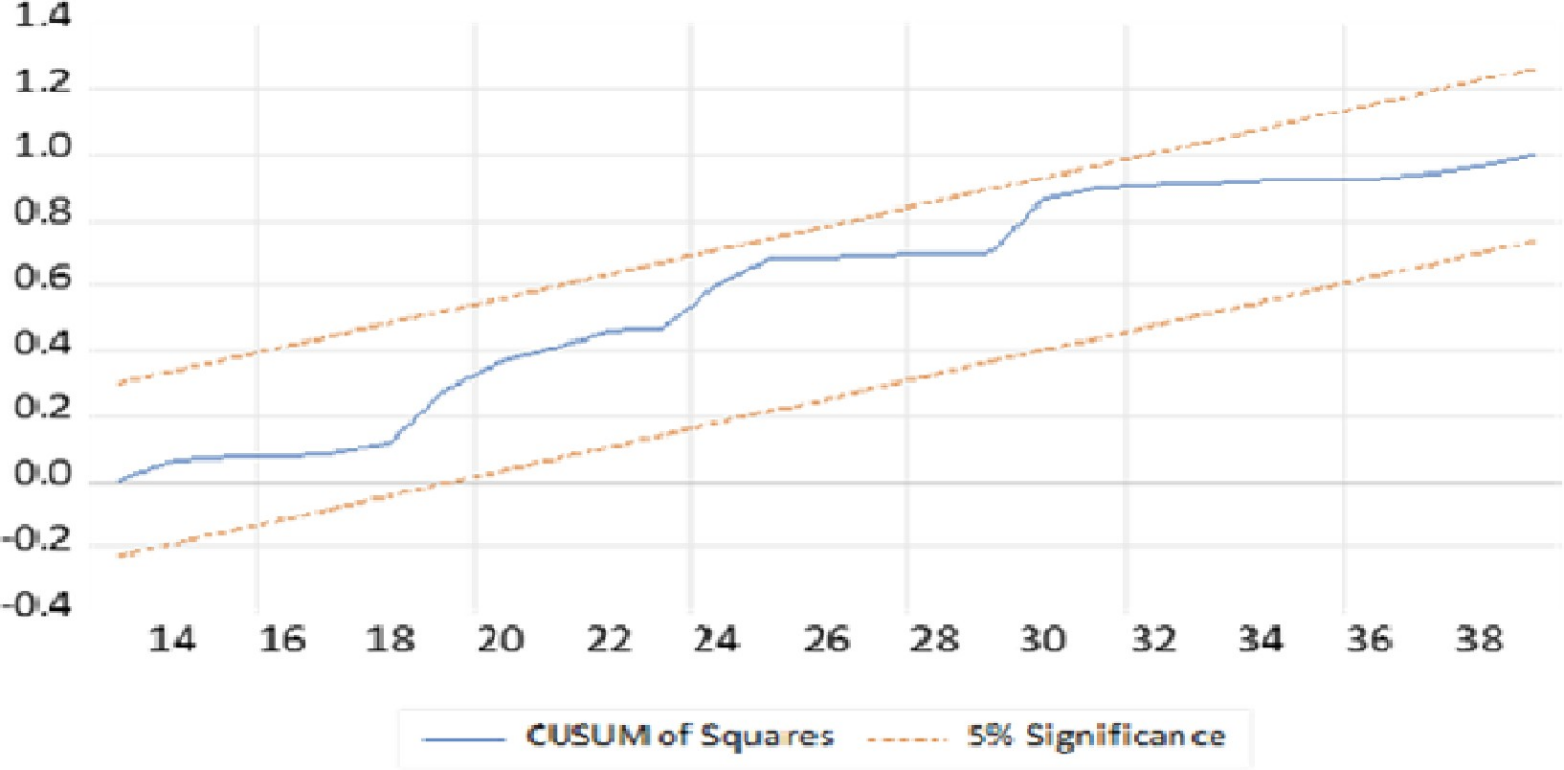
Cumulative sum of squared recursive residuals. CUSUM of Squares.

Moreover, as most time sires models are highly sensitive to structural breaks and unpredicted shocks in the worlds of open economy, tests for structural stability are crucial. To assess the long-run relationship and reliability of the trade balance and the covariates, the cumulative sum of recursive residuals (CUSUM) and the cumulative sum of squared recursive residuals (CUSUMSQ) are employed in this study as [54]. As a result, as we can see from Figs 3 and 4, the cumulative sum of recursive residuals and the cumulative sum of squared recursive residuals are within the critical bound of the 5% significance level respectively. Therefore, the long-run relationship or structure of the model is stable.

## 5. Conclusion and policy implications

This study investigate the asymmetric effect of real exchange rate on the trade balance of Ethiopia. Most of the findings in Ethiopia assume the effect of the exchange rate on the trade balance is symmetric, the implication is that an equal level of exchange rate depreciation or appreciation affects the trade balance by the same proportion, regardless of the direction of the impact. However, contemporary researchers challenge this conclusion from the ground that the effect of the devaluation on trade balance can be asymmetric. To fill this gap, this study adopts the non-linear autoregressive distributed lag model (NARDL) to verify the asymmetric effect of the real exchange rate on the trade balance of Ethiopia from 1992 to 2022. To the best of the author knowledge, this study is the first in exploring the issue in Ethiopian context.

The regression result disclosed that exchange rate devaluation/depreciation has a significant and asymmetric positive effect on the trade balance both in the long-run and short-run. Specifically, the exchange rate depreciation has a positive and significant effect on the trade balance by 0.843% and 0.856% in the long-run and short-run respectively following a unit change in the exchange rate. Moreover, appreciation of the real exchange rate (the negative component as indicated in the model) has also a positive and significant asymmetric effect on the trade balance by 15.07979% and 15.02%, on average, in the long-run and short-run respectively following a unit change in the exchange rate. Thus, while exchange rate depreciation is typically associated with improving the trade balance, appreciation can also have a positive effect under certain conditions. The outcome may depends on factors like the structure of the economy, the elasticity of demand for imports and exports, the nature of traded goods, and the role of inflation or debt in the economy. Therefore, the real-world dynamics can differ from theoretical assumptions. The implication is that understanding the specific context of a country’s trade relations and economic structure is key to determining how exchange rate volatility might affect its trade balance.

The major limitations encountered is that the study measures exchange rate of Ethiopia relative to the US dollar irrespective of considering the currency of other trading partners because of constraints of data availability, moreover, the study utilized the entire levels of both export and import volume indexes to measure trade balance, this may cause aggregation bias since it was not considered at disaggregated or commodity level.

Therefore, for future researchers, it is advisable to investigate the effect of exchange rate asymmetries on trade balance of Ethiopia at commodity level. The author further advises researchers to utilize comprehensive models like the dynamic stochastic general equilibrium (DSGE) models to largely extract the different passes-through effects of exchange rate on the trade balance at a disaggregated level.

It is a crucial policy insight for policymakers to understand exchange rate depreciation is not the only issue that matters to improve the trade balance, but it can also be improved when there is exchange rate appreciation (it creates understanding about the advantage of exchange rate volatility in Ethiopia). It is better to consider the non-linear model results and other exchange rate passes-through approaches to understand asymmetric effects before policy prescriptions are applied. Moreover, policy makers in Ethiopia should apply tight monetary policy rules and other parallel supplementary policy actions immediate to the policy change.

## Supporting information

S1 Dataset Raw data used in study analysis. (XLSX)
